# Does sevoflurane add to outpatient procedural sedation in children? A randomised clinical trial

**DOI:** 10.1186/s12887-017-0838-4

**Published:** 2017-03-24

**Authors:** Hugo Sérgio de Oliveira Gomes, Heloisa de Sousa Gomes, Joji Sado-Filho, Luciane Rezende Costa, Paulo Sucasas Costa

**Affiliations:** 1 Universidade Federal de Goiás, Goiânia, Brazil; 2 Dentistry Graduate Program, Universidade Federal de Goiás, Goiânia, Brazil; 3 University Hospital, Universidade Federal de Goiás, Goiânia, Brazil; 4 Faculty of Dentistry, Universidade Federal de Goiás, Goiânia, Brazil; 5 Department of Paediatrics, Universidade Federal de Goiás, Faculdade de Medicina, Rua 235 com Primeira Avenida, sem número, Setor Universitário, Goiânia, CEP 74605-020 Brazil

**Keywords:** Conscious sedation, Midazolam, Ketamine, Sevoflurane, Anti-anxiety agents, Child behaviour, Drug-related side effects and adverse reactions, Pain management, Dental anxiety, Dental care for children

## Abstract

**Background:**

There is little evidence concerning the effect of sevoflurane in outpatient procedural sedation, especially in children. We hypothesised that the addition of sevoflurane to a sedation regimen improves children’s behaviour with minimal adverse events.

**Methods:**

This is a randomised, triple-blind clinical trial conducted on an outpatient basis. Participants were 27 healthy children aged 4 to 6 years, who previously refused dental treatment with non-pharmacologic methods. All participants received oral midazolam (0.5 mg/kg, maximum 20 mg) and oral ketamine (3 mg/kg, maximum 50 mg) and, in addition: Group MK – 100% oxygen; Group MKS – inhalational sevoflurane at a sedative dose (final expired concentration between 0.3 and 0.4%). Dental appointments were video recorded for assessment of the children’s sleep patterns, crying, movements, and overall behaviour during the procedure with the Houpt scale. Intra- and post-operative adverse events were systematically reported. Data were analysed by bivariate analyses in the IBM SPSS v. 19, at a significance level of 5%.

**Results:**

MK (*n =* 13) and MKS (*n =* 14) did not differ regarding the Houpt scores (*P >* 0.05), but 53.8% of children in the MK group showed hysterical and continuous crying at the time of the local anaesthesia injection, compared to 7.1% of children in the MKS group (*P =* 0.01; phi = 0.5). There was a trend toward less crying and movement over time during the dental appointment in the MKS group (*P =* 0.48). Minor adverse events were observed in 10 MK children and 4 MKS children (*P =* 0.01).

**Conclusions:**

The addition of sevoflurane to oral midazolam-ketamine improved the children’s crying behaviour during local anaesthetic administration, and did not increase the occurrence of adverse events.

**Trial registration:**

Clinical Trials NCT02284204. Registered 5 October 2014.

## Background

Paediatric sedation outside of the operating room is a growing theme in the anaesthesiology field. Few new sedatives have been launched in the last decade, which suggests the need for further development of new routes and methods for delivery of existing anaesthetic agents [1].

Sevoflurane, “a near-perfect anaesthetic,” has been used for procedural sedation in an outpatient basis and in intensive care units [2]. However, there is little evidence of its effectiveness and safety in the paediatric population for a variety of procedures, including color Doppler ultrasound examination [3], voiding cystourethrography [4], gastrointestinal endoscopy [5], placement of central catheter in neonatal intensive care [6], magnetic resonance imaging [7], and dental treatment [8–13].

In paediatric dentistry, inhalation sedation with sevoflurane and nitrous oxide has reached success rates varying from 80 to 92% [8, 9, 12], which are increased to up 99% when intravenous midazolam and fentanyl are added as an alternative to general anesthesia [10, 11, 13]. A systematic review concluded that there is a need for clinical trials on sedation agents for dental treatment since only the agents that showed some (weak-very weak) evidence of effectiveness were oral midazolam and nitrous oxide inhalation [14].

Among the aforementioned studies, there were only 3 clinical trials conducted in the dental setting [9, 10, 12]; however, these studies did not examine the effects of sevoflurane as the sole gas inhaled anaesthetic agent (without nitrous oxide). Additional information is needed to add to the development of the outpatient sedation technique with sevoflurane. Thus, we performed this clinical trial with the aim of assessing the effect of sevoflurane during dental treatment in children aged 4 to 6 years old. We hypothesised adding sevoflurane to a sedation regimen would improve children’s behaviour with minimal adverse events.

## Methods

### Study design

This is a triple-blind, controlled, parallel-group clinical trial with balanced randomisation (1:1), approved by the Research Ethics Committee of the Universidade Federal de Goiás (UFG), Brazil (protocol #307/11). Children’s parents were informed about the study’s aims, procedures, benefits and risks, and invited to sign the consent form. This clinical trial was registered in the clinicaltrials.gov database with the number NCT02284204. Two sedative regimens were compared: midazolam and ketamine (MK), and midazolam, ketamine and sevoflurane (MKS). A pilot study with 10 children tested the operational aspects of this trial, and no changes in methods were needed after trial commencement.

### Participants screening, consent and preparation

Participants were children referred for dental treatment requiring sedation in the Dental Sedation Centre (*Núcleo de Estudos em Sedação Odontológica - “NESO”*) of the UFG Dental School. This centre is a community extension project where a multi-professional team (anaesthesiologist, paediatrician, paediatric dentist and psychologist) assist underserved children that need dental sedation to have their oral rehabilitation. The researchers follow the dental sedation protocol recommended by international guidelines, aiming to provide minimal to moderate sedation level [15]. The use of ketamine, however, can produce a dissociative sedation, which is defined as “a trancelike cataleptic state induced by the dissociative agent ketamine, characterized by profound analgesia and amnesia, with retention of protective airway reflexes, spontaneous respirations, and cardiopulmonary stability” [16].

To be included in this trial, children had to meet the following inclusion criteria: 4 to 6 years old, American Society of Anesthesiologists Physical Status I or II, presenting patent airway and effective nasal breathing, need of a restorative procedure in a lower primary molar, and negative behaviour in a previous attempt of dental treatment. Exclusion criteria included the following: previous experience of dental treatment under sedation, or having completed 7 years at the day of the dental sedation appointment.

After a child was considered eligible to participate in this trial and the parent signed the consent form, one of the researchers that did not take part in the interventions enrolled the child and scheduled the intervention appointment at the most convenient day for the parent. The child was then randomly assigned to either the MK or MKS group. Simple randomisation with a 1:1 allocation ratio was used. One researcher that did not participate in the interventions and outcomes assessments created a computer-generated list through the website Randomization.com (http://www.randomization.com). Each child was assigned to a group at the day of the intervention according to the consecutively numbered code generated in the list. As only the physicians knew the codes, they assigned participants to interventions.

### Dental sedation facility, personnel and monitoring

The sedation procedures were entirely carried out in an outpatient clinic, which accommodates open operatories to support medication administration, dental treatment and post-anaesthesia recovery.

The sedation team trained to comply with this protocol were: the paediatrician, one of the three anaesthesiologists, one of the two paediatric dentists, one dental hygienist and one observer responsible to monitor appropriate physiologic parameters.

The monitoring devices were: Infinity® Delta multiparameter monitor (Drägerwerk AG & Co., Lübeck, Germany) to assess oxygen saturation, electrocardiography, end-tidal carbon dioxide and anaesthetic gas (sevoflurane) analysis, and the defibrillator DEA Life400 Futura (Cmos Drake, Nova Lima, Brazil). Also, there was an on-site emergency cart that contains age-and size-appropriate equipment and drugs as recommended to resuscitate a nonbreathing and unconscious child [15].

### Dental sedation procedures

On the day of treatment, children arrived at the clinic to have their health status and fasting protocols checked, and then were accompanied by a parent and a trained observer to a sedative delivery place where the anaesthesiologist or paediatrician assessed the child’s vitals, prepared medications in a disposable syringe, and administered the drugs through the oral route: Midazolam 0.5 mg/kg, maximum dose 20 mg (Dormire®, Cristália, São Paulo, Brazil) and ketamine 3 mg/kg, maximum dose 50 mg (S+ Ketamin®, Cristália, São Paulo, Brazil). The observer monitored children’s behaviour and vital signs from since before sedative administration until discharge.

After 15 min, the trained observer and the child/parent dyad went to the place prepared for dental treatment and, according to the group assigned by the anaesthesiologist, the child received only oxygen (MK) or a mixture of oxygen and sevoflurane (MKS) provided through an anaesthesia workstation - Fabius® Plus (Drägerwerk AG & Co., Lübeck, Germany). The gases were provided through a mask placed over the nose of the child - Dynomite® Nasal Hood (Matrx-Parker Instrument, Hatfield, United States of America) - and analysed using an anaesthetic gas analyzer - Vamos® (Drägerwerk AG & Co., Lübeck, Germany). Initially, the child received 100% oxygen at a flow rate of 5 L/min, for 5 min. After this period, if the child was randomised to the MKS Group, sevoflurane was added in an initial concentration of 0.1%, with a 0.1% increment every 30 s until reaching a final expired concentration between 0.3 and 0.4%. In cases where the child had been randomised to the MK group (without sevoflurane), the anaesthesiologist simulated the supply of sevoflurane, but the child received only 100% oxygen. The gas analyzer screen was camouflaged by a strategic coverage, allowing the sevoflurane measures viewed only by the anaesthesiologist. The anaesthesiologist helped by the observer continuously monitored oxygen saturation, heart rate, fractional inspired carbon dioxide and end-tidal carbon dioxide.

**Fig. 1:**
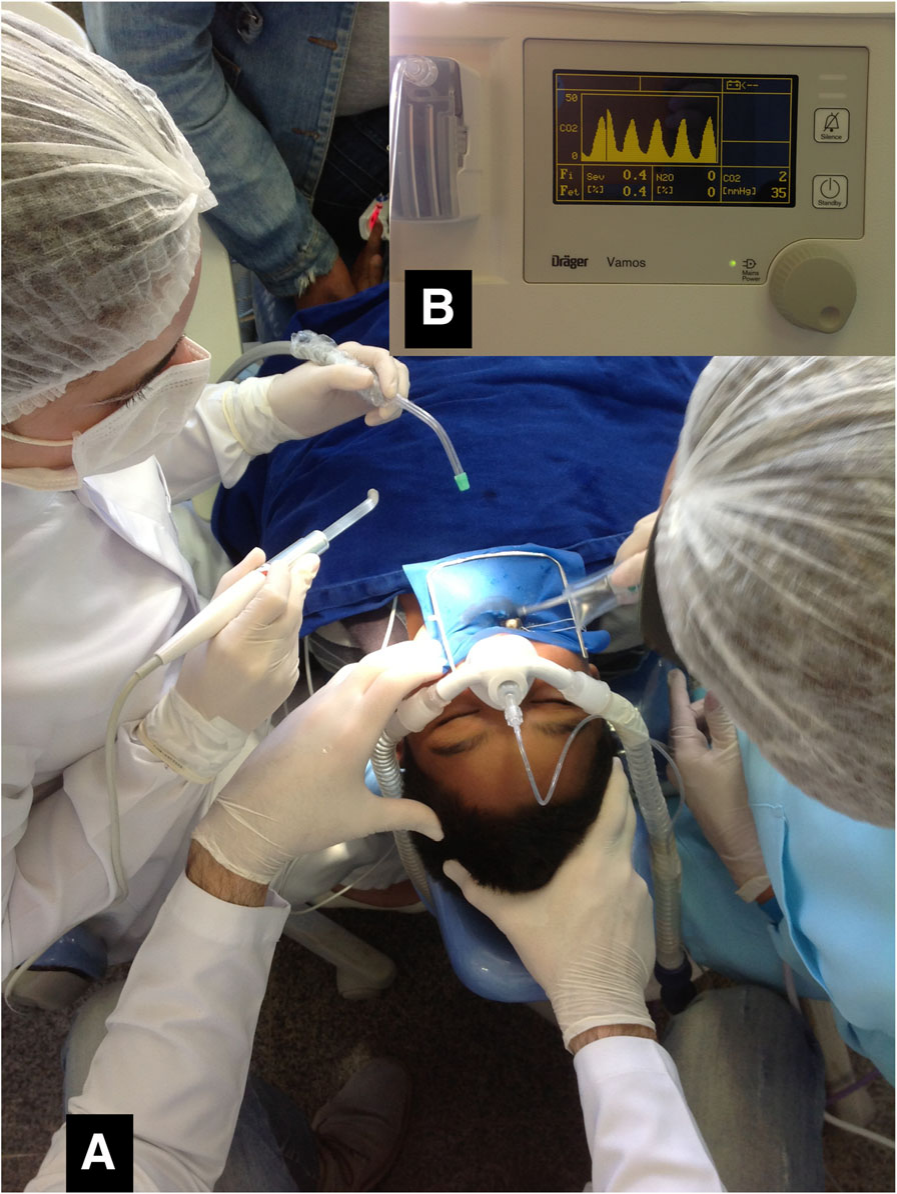
Sedation of children for dental treatment with sevoflurane provided through a nasal mask (**a**) and assessed by an anaesthetic gas analyser (**b**). Consents to publish this image were collected from the child’s parents and health professionals

After 15 min of placing the nasal mask and supply of gases, the paediatric dentist began the dental treatment as previously planned (Fig. 1 - consents to publish this image were collected from the child’s parents and health professionals). One certified paediatric dentist performed dental restorations after local anaesthesia (inferior alveolar nerve block with lidocaine 2% with epinephrine 1:100,000) and rubber dam isolation, using non-pharmacological tecnhiques in addition to the sedatives. A parent stayed with the child during the whole treatment.

At the end of the dental procedure, the dedicated observer, child and parent were to the post-anaesthetic recovery place and the child was monitored until the discharge criteria were met [15]: Satisfactory and stable cardiovascular function and airway patency; adequate state of hydration; child awake or easily arousable, with intact protective reflexes, can talk and sit up unaided. Post-operative recommendations (written protocol) were explained and given to parents/guardians.

The paediatric dentist, the child/parent, the observers and the data analyst were blinded to the allocation. Only the anaesthesiologist and the paediatrician knew the group that each child was assigned to properly manage any adverse event as needed.

### Behaviour assessment

The primary endpoint of this research was to evaluate the effects of sevoflurane on the behaviour of children aged 4 to 6 years old during dental treatment by comparing 2 sedative regimens.

The dental sedation appointment was video recorded with a digital camera to allow a detailed observation of children’s behaviour during the dental treatment.

The child’s behaviour was evaluated using the Houpt scale [17], with an observer blinded to the sedation group that was previously trained and calibrated. The Houpt scale is a tool used to assess a child’s behaviour during dental sedation according to specific categories and scores including: sleep (1-awake, 2-drowsy, disoriented), movement (1-violent, 2-continuous, 3-controllable, 4-no movement), cry (1-hysterical, 2-continuous, 3-intermitent, 4-no cry), and overall behaviour (1-treatment aborted, 2-poor, 3-regular, 4-good, 5-very good, 6-excellent). This scale has been reported in various paediatric sedation studies and has good psychometric properties [18, 19]. During the sessions, both groups had the children's behaviour evaluated every minute and also in 3 specific moments of the dental treatment: at the time of anaesthesia; at the time of use of high-speed handpiece, and at the end of the treatment. In addition, an overall assessment of behaviour during the treatment session was conducted.

A senior researcher with expertise in paediatric dentistry trained 4 observers (dental students) to assess the children’s behaviour in the digital videos in a four-hour workshop with videos of children that did not take part in this study. Those observers were not present at the moment of sedation in order to not smell any eventual gas (sevoflurane). For calibration, each observer watched five-minute videos of 3 patients during the clinical examination and registered a specific score for sleep, movement, cry and overall behaviour for each minute of the video. Weighted kappa values were between 0.8 and 0.9 for intra- and inter-examiner agreement.

### Adverse events assessment

The secondary endpoint was the occurrence of adverse events in the perioperative period and in the first 24 h after dental treatment sessions under sedation.

The World SIVA International Sedation Task Force proposed a definition that is specific to sedation-related occurrences: “unexpected and undesirable response(s) to medication(s) and medical intervention used to facilitate procedural sedation and analgesia that threaten or cause patient injury or discomfort” [20]. The Task Force presented a tool for reporting adverse sedation events. This tool was used to develop specific forms of possible adverse events for this research [20].

An observer was responsible for completing the specific forms for possible adverse events in the intraoperative period and in the 24 h following the dental sedation appointment (this information was obtained by telephone contact with parents/guardians).

### Statistical planning and analysis

Sample size was estimated based on a pilot study of 10 children that were included in the final sample. Diverse calculations considering 3 different outcomes were performed: occurrence of continuous or hysterical crying during the procedure (MK = 50.0% and MKS = 0%); occurrence of continuous or violent movement during the procedure (MK = 33.3% and MKS = 0%), and occurrence of any adverse event in the first 24 h post-operative period (MK = 83.3% and MKS = 25.0%). For each outcome, a sample size of 11, 18 or 10 children, respectively, was established to achieve a probability of 80% to detect a difference in the level of significance of 5% in a two-tailed hypothesis test.

Data were analysed using the IBM SPSS version 19.0 (SPSS Inc. Chicago, IL, USA). MK and MKS groups were compared in terms of the dependent variables including the child's behaviour (movement, cry, overall), vital signs (heart rate and oxygen saturation) and occurrence of adverse events (intraoperative and the first 24 h postoperative). Independent variables included the child’s gender, age, weight, dose of oral sedatives, need of physical restraint, duration of the dental sedation appointment, heart rate and oxygen saturation.

After testing for data distribution in each continuous variable, data were analysed through bivariate tests: Student’s *T*-Test, Mann-Whitney’s *U*-Test, Fisher’s Exact-Test, Pearson’s Chi-Square and Likelihood-Ratio Chi-Square. The significance level was set at *P <* 0.05.

## Results

**Fig. 2:**
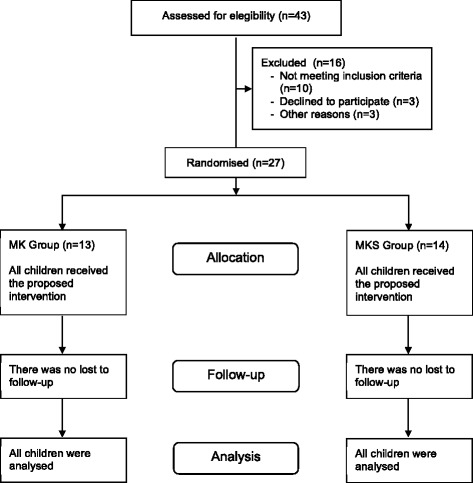
Flow diagram of the progress through the clinical trial phases

From a total of 43 children accessed for eligibility during 2014, 27 children (nine girls and 18 boys) with a mean age 4.9 years old (standard deviation “SD” 0.8) met the inclusion criteria and received the interventions. Such children were randomised as follows: 13 children (10 boys) for the MK Group and 14 children (8 boys) for the MKS Group (Fig. 2). No participants were excluded after randomization, and all randomised children were included in the analysis. Groups did not differ regarding the demographic data or characteristics of the dental treatment sessions (Table 1). All children completed the dental treatment as planned. The sedation level varied among minimal, moderate and dissociative status.

**Table 1:** Demographic data and characteristics of the dental treatment sessions of children

Variables	n (%), mean (SD) or median (25%–75%)		P
	MK Group (*n =* 13)	MKS Group (*n =* 14)	
Gender (n)			0.28^a^
Male	10 (76.9%)	8 (57.1%)	
Female	3 (23.1%)	6 (42.9%)	
Age (years)	4.7 (0.6)	5.2 (0.8)	0.07^b^
Weight (kg)	16.5 (15.7–19.6)	19.6 (16.7–23.9)	0.18^c^
Dose of oral sedatives			
Ketamine (mg)	48.6 (2.3)	48.4 (3.4)	0.54^a^
Midazolam (mg)	8.9 (1.2)	9.5 (3.1)	0.32^b^
Need of physical restraint			
During administration of local anaesthesia	9 (69.2%)	8 (57.1%)	0.70^a^
During use of high-speed handpiece^d^	7 (58.3%)	8 (57.1%)	0.86^a^
Dental procedure duration (min)	44.6 (8.2)	45.0 (6.3)	0.89^b^
Time for recovery (min)	84.7 (24.3)	72.2 (23.4)	0.23^b^

*SD* Standard deviation, *MK* midazolam-ketamine, *MKS* midazolam-ketamine-sevoflurane

^a^Pearson’s Chi-Square

^b^Student’s *T*-Test

^c^Mann-Whitney’s *U*-Test

^d^High-speed handpiece was not used in one MK child because of severe uncooperativeness

**Fig. 3:**
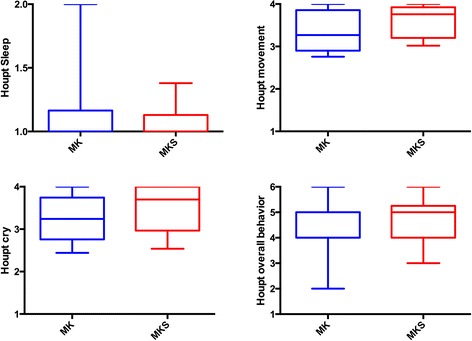
Behaviour scores according to Houpt scales comparing groups midazolam/ketamine (MK) and midazolam/ketamine/sevoflurane (MKS) (*P >* 0.05; Mann-Whitney test)

**Fig. 4:**
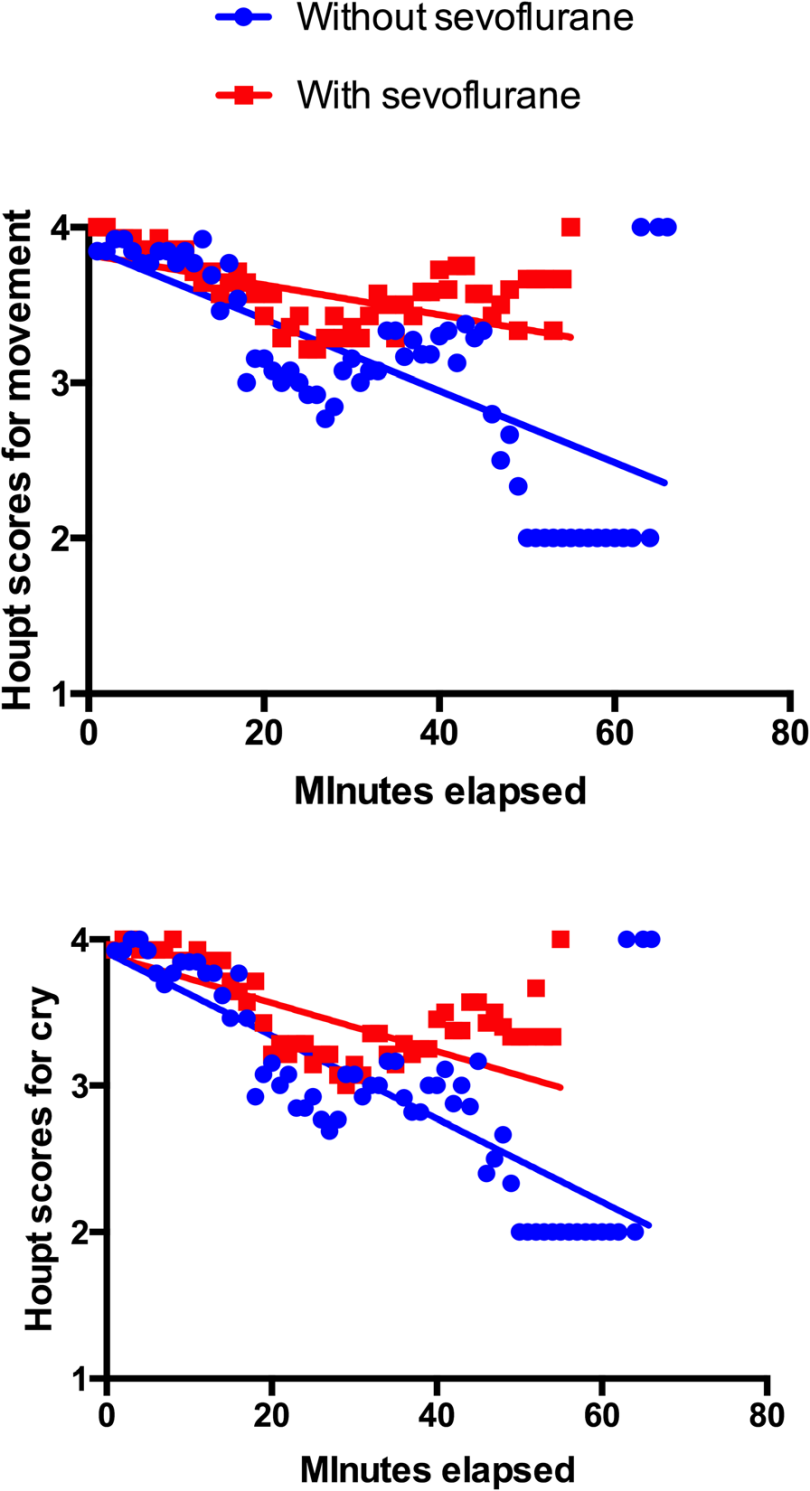
Houpt scores for “Movement” and “Cry” during the dental treatment sessions under sedation, according to the midazolam/ketamine (MK) and midazolam/ketamine/sevoflurane (MKS) group

Children’s behaviour, according to Houpt scale scores, did not differ between the MK and MKS groups, considering the median scores for sleep, movement and cry in the whole session (Fig. 3), or at specific times of dental treatment (Table 2). For the majority of assessments, children were awake, with absent or minimal crying, or movement; overall behaviour was mostly good to very good. An additional analysis was performed to compare the groups and children presenting more negative scores (3 and 4) for crying and movement; MKS children showed less hysterical/continuous crying at the time of local anaesthesia administration (Table 3). By plotting the evolution of the children’s crying and movement during the dental session, we observed that children in the MKS group tended to show a smaller drop in Houpt scores (Fig. 4).

**Table 2:** Houpt scores observed at different times of the dental treatment session while under sedation

Houpt Scores	Median (25%–75%)		P^a^
	MK Group (*n =* 13)	MKS Group (*n =* 14)	
Sleep			
Administration of anaesthesia	2.0 (1.0–2.0)	2.0 (2.0–3.0)	0.26
Use of high-speed handpiece^b^	2.0 (1.3–2.0)	2.0 (2.0–2.3)	0.81
End of session	2.0 (1.5–2.0)	2.0 (2.0–2.0)	0.33
Movement			
Administration of anaesthesia	3.0 (2.0–3.5)	4.0 (2.8–4.0)	0.69
Use of high speed^b^	3.0 (1.3–4.0)	3.5 (2.0–4.0)	0.50
End of session	4.0 (2.0–4.0)	4.0 (2.0–4.0)	0.12
Cry			
Administration of anaesthesia	2.0 (2.0–4.0)	3.0 (3.0–4.0)	0.18
Use of high-speed handpiece^b^	2.5 (1.3–4.0)	3.5 (2.0–4.0)	0.45
End of session	3.0 (2.5–4.0)	4.0 (2.0–4.0)	0.10

*MK* midazolam-ketamine, *MKS* midazolam-ketamine-sevoflurane

^a^Mann-Whitney’s *U*-Test

^b^High-speed handpiece was not used in one MK child because of severe uncooperativeness

**Table 3:** Hysterical/Continuous Cry and Violent/Continuous Movement at specific moments of the local anaesthesia, use of high rotation and at the end of the procedure

Variables	n (%)		P	Effect size (*phi*)
	Group MK (*n =* 13)	Group MKS (*n =* 14)		
Hysterical/Continuous Cry				
Administration of local anaesthesia	7 (53.8%)	1 (7.1%)	0.01^a^	0.5
Use of high speed^c^	5 out of 12 (41.7%)	5 (35.7%)	0.76^b^	0.2
End of session	5 (38.5%)	3 (21.4%)	0.42^a^	0.2
Violent/Continuous Movement				
Administration of local anaesthesia	4 (30.8%)	1 (7.1%)	0.17^a^	0.3
Use of high speed^c^	4 out of 12 (33.3%)	2 (14.3%)	0.37^a^	0.2
End of session	4 (30.8%)	1 (7.1%)	0.17^a^	0.3

*MK* midazolam-ketamine, *MKS* midazolam-ketamine-sevoflurane

^a^Fisher’s Exact-Test

^b^Pearson’s Chi-Square

^c^High-speed handpiece was not used in one MK child because of severe uncooperativeness

Oxygen saturation and pulse remained adequate throughout the procedure and did not differ between groups (Table 4).

**Table 4:** Heart rate and oxygen saturation observed at different times of the dental treatment session while under sedation

Physiologic signs	Mean (SD) or median (25%-75%)		P
	MK Group (*n =* 13)	MKS Group (*n =* 14)	
Heart rate (beats per minute)			
Reception (baseline)	96.4 (17.7)	94.3 (17.2)	0.77^a^
Administration of anaesthesia	122.7 (28.6)	107.1 (22.3)	0.13^a^
Use of high speed^c^	125.2 (32.9)	124.6 (26.9)	0.96^a^
End of session	133.2 (25.9)	123.9 (24.7)	0.35^a^
Oxygen saturation (%)			
Reception (baseline)	97.5 (95.3–98.0)	97.5 (96.3–98.0)	0.89^b^
Administration of anaesthesia	99.0 (98.0–100)	100.0 (99.0–100)	0.12^b^
Use of high speed^c^	99.5 (97.3–100)	99.0 (98.0–100)	0.89^b^
End of session	100.0 (98.0–100)	99.0 (97.5–100)	0.42^b^

SD: Standard deviation; MK: midazolam-ketamine; MKS: midazolam-ketamine-sevoflurane

^a^Student’s *T*-Test

^b^Mann-Whitney’s *U*-Test

^c^High-speed handpiece was not used in one MK child because of severe uncooperativeness

Intraoperatively, the only one case of adverse event observed and classified as minor risk was “failed sedation”, i.e., the “inability to attain suitable conditions to humanely perform the procedure” [20]: one girl from the MK group had the dental treatment aborted after the administration of local anaesthesia because of her severe uncooperativeness; she did receive a tooth restoration with glass ionomer cement instead of composite. Prolonged recovery (Table 1) as well as serious adverse events, such as severe drop oxygen saturation (<75% or <90% for more than 60 s), prolonged apnea (>60 s), cardiovascular shock or cardiac arrest/pulse absence were not observed. There was no deep sedation at any time of evaluation.

Of the total sample, 51.9% (14 children) reported adverse events in the 24 h after sedation including 10 MK children and 4 MKS children (*P =* 0.01, Likelihood-Ratio Chi-square; effect size 0.3). The most frequently observed adverse events were excessive drowsiness (22.2%, 6 children) and vomiting (22.2%, 6 children). Nausea was observed in 3 children (14.3%); more than one adverse event may have been reported for the same patient. Other adverse events observed in 24 h after sedation included restlessness, dizziness, malaise, headache and otalgia (data not shown). One MK child had vomiting in the perioperative period. No children had to be hospitalized after discharge.

## Discussion

The main finding of this study was that the addition of inhaled sevoflurane to an oral midazolam/ketamine regimen did not improve the overall behaviour of the children undergoing sedation for dental treatment (except during local anaesthesia administration), but did result in less minor adverse events than the oral regimen alone. The present outcomes on sevoflurane sedation success were less favorable than previous clinical trials [9, 10, 12]; methodological aspects should explain these differences and will be discussed.

First, we did not associate sevoflurane to nitrous oxide as others did previously [9, 10, 12]; nitrous oxide could add a second gas effect to sevoflurane and improve sedation [21]. Second, both groups in the present study received an association of midazolam and ketamine, which has been proved effective in moderate sedation in both pediatric and dental settings [22, 23]. Other trials associated sevoflurane/nitrous oxide with intravenous midazolam [10], or only compared sevoflurane/nitrous oxide with nitrous oxide [9, 12]. Third, the participants in our study were restricted to an age range of 4–6 years old, whilst other trials included older children ranging from 3–10 [9], 6–14 [10], and 6–15 [12] years old. The younger age range of children in our study presented a cognitive development issue that would hinder their cooperation with a moderate, interactive sedation, compared with older children. Fourth, we analysed the children’s behaviour minute by minute throughout the dental session, however other trials [9, 10, 12] considered the completion of dental treatment as the main outcome.

In addition, there was a difference in sample size between our study and the aforementioned trials. In one trial, the sample size was 30 [12], whilst other two trials included more than 400 [9] and approximately 700 children [10]. As effect sizes observed in this study varied from small to large, one cannot affirm that sevoflurane does not improved children’s behaviour outcomes. On the other side, our limited sample size was controlled in respect to the procedure performed, the same type of local anaesthesia, a single trained operator, use of calibrated observers for the main outcome (child behaviour), and children within a limited age range with similar baseline behaviour. Thus, future studies would benefit from larger sample sizes and to control for potential bias involved in a procedural sedation.

Nevertheless, children that did not receive sevoflurane in this study showed more continuous/hysterical crying during the local anaesthesia administration (*P =* 0.01, large effect size). We speculate that sevoflurane could have prevented pain from the injection. A trend was observed in sustained control of behaviour in patients using sevoflurane for sedation, comparing to those without the drug. Other studies link increased effectiveness at the completion of treatment with the addition of sevoflurane for sedation [9, 10, 24].

In this study, there was a need to raise the fraction of inspired sevoflurane to levels greater than 1% in order to obtain the final expired concentration approximately 0.3–0.4% as in the other study [25]. It is speculated that the movement of the head of the child, the difficulty of nasal mask seal, and the presence of mouth breathing are factors involved in a possible dilution of the fraction of inspired sevoflurane; factors that could have minimised the benefit in behavioural control through the addition of inhalational anaesthetic while under sedation. Actually, the range of concentration for sevoflurane sedation has not yet been defined, varying from 0.3% (offered) [24] in dental sedation to 4.0% [26]. We used the goal of 0.3% to 0.4% end tidal concentration. In this group of children, sevoflurane used as a sedative was associated with minor adverse events in a few cases. However, a device for its delivery outside the operating room is not yet standardised [24]. The Anaesthetic Conserving Device (AnaConDa®) has being used to deliver sevoflurane to sedate mechanically ventilated patients through traqueal cannula [27–29]. In this study, sevoflurane was provided through a mask placed over the nose; some authors used a nasal cannula [25] or the same mask used to deliver nitrous oxide [9, 10, 30].

On the other hand, like another trial [12], we did not find any serious adverse events associated with the use of sevoflurane; besides, it did not associate with longer recovery or post-discharge readmission. Furthermore, there was a higher incidence of adverse events in the group that did not use sevoflurane. To the best of our knowledge, the literature does not explain the beneficial effect of sevoflurane in the postoperative period regarding the occurrence of minor adverse events, and should be further investigated in studies with larger samples and different settings.

This randomised clinical trial contributes to the evidence for the efficacy of inhaled sevoflurane as a sedation agent. Yet, the issues discussed indicate that there are still limitations for extensive clinical use of sevoflurane in paediatric anaesthesia. Furthermore, it should be emphasised that more complex sedation techniques, such as sevoflurane sedation, should be performed in specialised centres by experienced teams [10].

## Conclusions

The addition of inhaled sevoflurane to an oral mixture of midazolam and ketamine did not significantly improve overall young children’s cooperation with dental treatment, except during the local anaesthetic administration. There was a trend that children who received sevoflurane cried and moved less than those who received oxygen. Besides, the sevoflurane supplement did not cause more adverse events.

## Abbreviations

MK: Midazolam and ketamine group
MKS: Midazolam, ketamine and sevoflurane group
NESO: *Núcleo de Estudos em Sedação Odontológica* (name of the dental sedation centre)
SD: Standard deviation
UFG: *Universidade Federal de Goiás* (name of the Brazilian university)
